# Chronic otorrhea and osteomyelitis of the external auditory canal by *Achromobacter xylosoxidans*: an uncommon diagnosis

**DOI:** 10.1007/s00405-024-08465-8

**Published:** 2024-02-17

**Authors:** Coloma Grau-van Laak, Carmen Ruiz-García, Luis Lassaletta, J. Manuel Morales-Puebla

**Affiliations:** 1 PhD Program in Medicine and Surgery, Autonomous University of Madrid, Madrid, Spain; 2 Department of Otolaryngology, La Paz University Hospital, Paseo de la Castellana, 261, 28046 Madrid, Spain; 3 IdiPAZ Research Institute, Madrid, Spain; 4 Biomedical Research Networking Centre on Rare Diseases (CIBERER), Institute of Health Carlos III, Madrid, Spain

**Keywords:** *Achromobacter xylosoxidans*, Otorrhea, Osteomyelitis, Chronic otitis externa

## Abstract

**Purpose:**

*Achromobacter xylosoxidans* is an emerging pathogen mainly associated with resistant nosocomial infections. This bacteria had been isolated in the ear together with other pathogens in cultures from patients with chronic otitis media, but it had never been reported as a cause of osteomyelitis of the external auditory canal.

**Case presentation:**

We present a unique case of a healthy 81-year-old woman who presented with left chronic otorrhea refractory to topical and oral antibiotic treatment. Otomicroscopy revealed an erythematous and exudative external auditory canal (EAC) with scant otorrhea. The tympanic membrane was intact, but an area of bone remodeling with a small cavity anterior and inferior to the bony tympanic frame was observed. Otic culture isolated multi-drug-resistant *A. xylosoxidans*, only sensitive to meropenem and cotrimoxazole. Temporal bone computed tomography showed an excavation of the floor of the EAC compatible with osteomyelitis. Targeted antibiotherapy for 12 weeks was conducted, with subsequent resolution of symptoms and no progression of the bone erosion.

**Conclusions:**

Atypical pathogens such as *A. xylosoxidans* can be the cause of chronic otitis externa. Early diagnosis and specific antibiotherapy can prevent the development of further complications, such as osteomyelitis. In these cases, otic cultures play an essential role to identify the causal germ. This is the first case of EAC osteomyelitis due to *A. xylosoxidans* reported to date.

## Introduction

*Achromobacter xylosoxidans* is a non-fermenting aerobic Gram-negative bacillus that can be found in aquatic environments and aqueous fluids, such as swimming pools, well-water, dialysis solutions, chlorhexidine solutions, and on plants [1]. It was first described in 1971 by Yabuuchi and Oyama, who isolated it in ear discharges from patients with chronic otitis media [2]. Since then, it has been identified in chronic otitis media effusion along with other pathogens, as mixed flora, as well as in various human body fluids, including respiratory tract secretions and peritoneal fluid [3]. Although it is considered an opportunist pathogen with low virulence, it can cause serious infections in the immunocompromised population [3, 4]. It is associated with nosocomial infections, being bacteremia, pneumonia and chronic cystic fibrosis lung infection the most common clinical presentations [3].

*Achromobacter xylosoxidans* infections are challenging due to their multidrug resistance. This pathogen is innate, strain-specific resistant to beta-lactams, aminoglycosides, fluoroquinolones, aztreonam, tetracyclines and cephalosporins [1]. To date, only one case of chronic otitis externa and chronic otomastoiditis caused by *A. xylosoxidans* has been reported [5]. In addition, several cases of osteomyelitis due to *A. xylosoxidans* have been described [6–14], but none of them affecting the ear.

We report a unique case of osteomyelitis of the external auditory canal (EAC) caused by *A. xylosoxidans* in an elderly immunocompetent woman.

## Case report

**Fig. 1:**
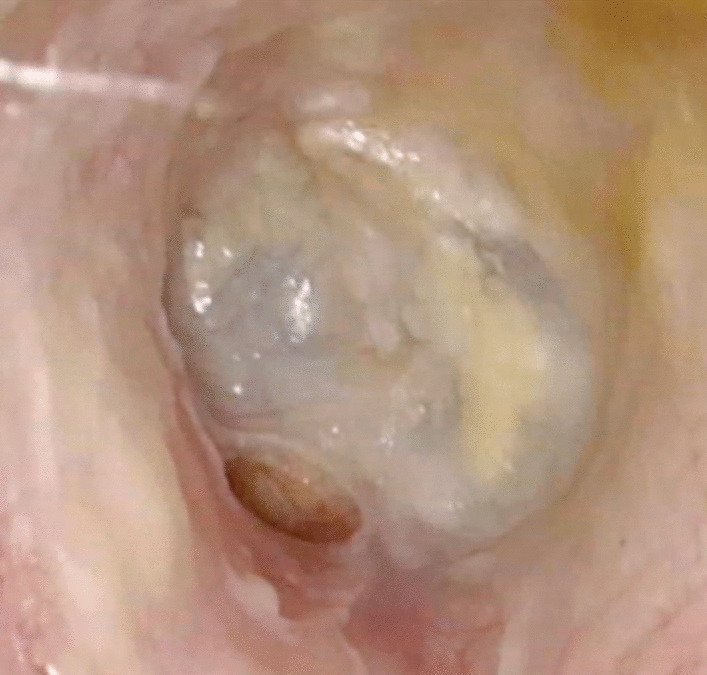
Otomicroscopy at diagnosis. Intact tympanic membrane with an area of bone remodeling anterior and inferior to the bony tympanic frame

An 81-year-old female with no significant medical history, presented to our Otolaryngology Department with a 2-month history of mild left otalgia, otic itching and otorrhea. During this period, she had been treated by her primary care physician without any improvement. She was prescribed oral amoxicillin 500 mg every 8 h for 1 week. As part of the topical treatment, she received ciprofloxacin, beclomethasone dipropionate/clioquinol, fluocinolone and dexamethasone/polymyxin B/trimethoprim. Each treatment was prescribed for 2 weeks. Otomicroscopy revealed erythema, edema and scant otorrhea in the anteroinferior part of the EAC. The tympanic membrane (TM) was intact. An area of bone remodeling with a small cavity anterior and inferior to the bony tympanic frame was observed, with accumulation of otorrhea in it. There was no evidence of granulation tissue or bone exposure (Fig. 1).

No fever or facial palsy was noticed. Leukocyte count was 5930/mm^3^ (3.6–10.5 /mm^3^), C-reactive protein was 4,6 mg/L (0–5 mg/L) and erythrocyte sedimentation rate (ESR) was 25 mm/h (0–30 mm/h). A culture of the otorrhea isolated multiresistant *A. xylosoxidans*, only susceptible to meropenem and cotrimoxazole (Table 1). No cultures for fungal infection were needed as *A. xylosoxidans* was identified as the causal pathogen in the first conventional culture.

**Table 1:** Antibiogram of *A. xylosoxidans* obtained from left ear discharge

Antibiotics	MIC (mcg/ml)	Susceptibility
Piperacilin/tazobactam	16	R
Cefotaxime	> 64	R
Meropenem	0.25	S
Cotrimoxazole	≤ 1/19	S

^Antibiogram interpreted with EUCAST breakpoints^

^*MIC*minimum inhibitory concentration, *S*susceptible, *R*resistant^

**Fig. 2:**
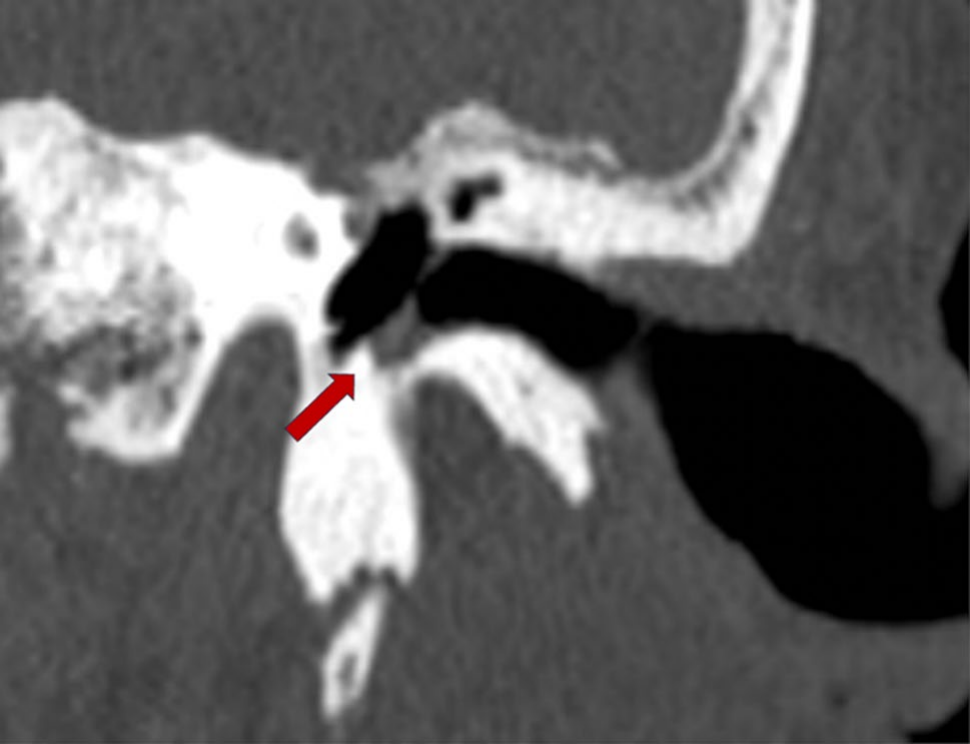
CT left temporal bone coronal plane. Excavation of EAC floor and tissue accumulation at the inferior insertion of the tympanic membrane (arrow)

Temporal bone computed tomography (CT) showed a thickening of the left TM with tissue accumulation at its lower insertion and excavation of EAC floor compatible with osteomyelitis (Fig. 2).

A multidisciplinary management approach was undertaken with the Infectious Disease Department. Given the compatible diagnosis of osteomyelitis, a treatment course of 6 weeks with intravenous meropenem was promptly started (2 g/8 h for the first week; 1 g/8 h for the next 5 weeks) followed by 4 weeks of oral cotrimoxazole (trimethoprim 160 mg/sulfamethoxazole 800 mg every 12 h). Topical antibiotic therapy (dexamethasone + polymyxin B + trimethoprim, 3–4 drops every 12 h) was continued throughout this time. She was hospitalized for the initial 3 weeks of treatment. Afterward, intravenous treatment was continued at home facilitated by our home hospitalization program. Symptomatology rapidly disappeared with treatment. Four months after completing the treatment, the patient remained asymptomatic. Upon otomicroscopy, the bony excavation in the lower tympanic frame persisted, stable and epithelialized, with no evidence of otorrhea or progression. A CT control also confirmed the absence of progression of the excavation of the EAC floor. Audiometry showed right normal hearing and left mild conductive hearing loss with a maximum speech discrimination of 100% in both ears, at 40 dB in the right ear and 50 dB in the left ear.

## Discussion

This report presents the case of a healthy 81-year-old woman who presented with osteomyelitis of the EAC due to multidrug resistant *A. xylosoxidans*. To the best of our knowledge, this is the first case of osteomyelitis of the EAC caused by this pathogen. While ten cases of osteomyelitis due to *A. xylosoxidans* have been reported in literature [6–14], none have affected the ear (Table 2).

**Table 2:** Summary of previously reported cases of osteomyelitis caused by *A. xylosoxidans*

Authors	Year	Localization	Gender	Age	Immunosuppression	Comorbidities	Coinfection
Dubey et al. [6]	1988	Tibia	Female	13	N/A	N/A	*Enterobacter agglomerans*
Hoddy et al. [7]	1991	Metatarsal	Male	11	None	None	None
Walsh et al. [8]	1993	Sternum	Female	55	None	Rheumatic heart disease, mitral valve replacement	None
Walsh et al. [8]	1993	Sternum	Male	65	None	Coronary artery disease	None
Stark et al. [9]	2007	Peroneal bone	Male	61	Good’s Syndrome	None	None
Ozer et al. [10]	2012	Calcaneus	Male	55	None	Foot drop, Squamous cell carcinoma	*Enterococcus faecium*
Fort et al. [11]	2014	L5–S1 vertebral bones	Female	29	None	None	*Propionibacterium acnes*
Pamuk et al. [12]	2015	Talus, Navicular, Cuneiform	Female	15	None	None	None
Shinha et al. [13]	2015	Hallux	Male	39	None	Diabetes	No
Imani et al. [14]	2021	Femur	Male	23	None	None	*Staphylococcus aureus*
Present case	2023	External auditory canal	Female	81	None	None	No

^*N/A*not available/not reported^

^Revision and extension of Table 2 in Imani et al. (14)^

The main differential diagnosis considered was malignant external otitis (MEO), as the clinical presentation was otalgia and chronic otorrhea. MEO usually affects elderly individuals with poorly controlled diabetes and/or immunosuppression [15]. However, our patient had no comorbidities and no evidence of immunosuppression. Another difference from MEO was the extent of the illness. MEO typically presents with extensive inflammation that progresses regionally from the EAC to the soft tissues and the bone, eventually involving the skull base [15]. In this case, mild inflammation was observed in the EAC, with bone erosion limited to a restricted area in the anteroinferior wall of the EAC.

The best imaging modality for the diagnosis and follow-up of temporal bone osteomyelitis is controversial. Traditionally, methylene diphosphonate (MDP)-technetium-99 m (^9^^9m^Tc) and Gallium-67 (^67^Ga) scans were standard for MEO diagnosis and management [16]. Recent meta-analysis and systematic reviews, such as the work by Moss et al., have challenged the reliability and efficacy of ^99m^Tc and ^67^Ga scans due to poor sensitivity, specificity, and limited ability to assess disease resolution [17]. Thus, many physicians now rely upon standard CT and magnetic resonance imaging (MRI) scans due their superior anatomical resolution in diagnosing and managing osteomyelitis [18]. CT imaging provided crucial insights in identifying thickening of the left tympanic membrane, tissue accumulation, and bony erosion, consistent with osteomyelitis in our patient. Hybrid nuclear studies, specifically ^18^F-FDG–PET/CT, have emerged as promising tools due to their higher sensitivity, specificity, cost-effectiveness, and reduced radiation exposure. However, further extensive studies are necessary to establish its definitive role in MEO diagnosis and follow-up [19]. Based on the clinical history and CT scan findings showing compatible signs of EAC osteomyelitis, a treatment plan was promptly initiated. The patient exhibited an early positive response, obviating the need for additional imaging studies for diagnostic purposes.

*Achromobacter* *xylosoxidans* has been related to nosocomial infections in immunocompromised patients [1, 3] and it is a frequent agent in humid environments [4]. Medical history of this patient did not reveal any immunologic disorder or chronic illness that could predispose to this infection. Neither a recent contact with humid environments was detected. Aging alters the immune system and decreases its ability to fight infections [20]. The advanced age of our patient could be considered as a predisposing factor for acquiring this infection. As seen in Table 2, all other cases of osteomyelitis due to *A. xylosoxidans* were much younger than our patient. It remains unclear how our patient contracted this disease.

## Conclusions

Chronic ear infection caused by *A. xylosoxidans* is uncommon and developing osteomyelitis is extremely rare. This is the first reported case of osteomyelitis of the EAC due to this organism. Otolaryngologists must be aware that atypical bacteria such as *A. xylosoxidans* can be responsible for chronic otitis externa. Otic cultures in chronic otorrhea play an essential role to identify the causal germ. Although *A. xylosoxidans* is considered an opportunistic bacteria with low virulence, its intrinsic resistance to a wide spectrum of antibiotics makes it difficult to eradicate. Early diagnosis and accurate treatment can prevent osteomyelitis progression and further related complications.

## Data Availability

The data presented in this study are available on reasonable request from the corresponding author.

## References

[1] Bonis BM, Hunter RC (2022). JMM Profile: *Achromobacter*
*xylosoxidans*: The cloak-and-dagger opportunist. J Med Microbiol.

[2] Yabuuchi E, Oyama A (1971). *Achromobacter*
*xylosoxidans* n. sp. from human ear discharge. Jpn J Microbiol.

[3] Schoch P, Cunha B (1988). Nosocomial *Achromobacter*
*xylosoxidans* infections. Infect Control Hosp Epidemiol.

[4] Sakurad A (2012). *Achromobacter*
*xylosoxidans* [*Achromobacter*
*xylosoxidans*]. Revista chilena de infectología: órgano oficial de la Sociedad Chilena de Infectología.

[5] Curry S, Richman E, Hatch J (2020). Chronic otomastoiditis and otitis externa caused by *Achromobacter*
*xylosoxidans*. Otolaryngol Case Reports.

[6] Dubey L, Krasinski K, Hernanz-Schulman M (1988). Osteomyelitis secondary to trauma or infected contiguous soft tissue. Pediatr Infect Dis J.

[7] Hoddy DM, Barton LL (1991). Puncture wound-induced *Achromobacter*
*xylosoxidans* osteomyelitis of the foot. Am J Dis Child (1960).

[8] Walsh RD, Klein NC, Cunha BA (1993). *Achromobacter*
*xylosoxidans* osteomyelitis. Clin Infect Dis.

[9] Jj S (2007). Alcaligenes xylosoxidans osteomyelitis without trauma in a patient with Good’s syndrome. Eur J Intern Med.

[10] Ozer K, Kankaya Y, Baris R, Bektas CI, Kocer U (2012). Calcaneal osteomyelitis due to *Achromobacter*
*xylosoxidans*: a case report. J Infect Chemother.

[11] Fort NM, Aichmair A, Miller AO, Girardi FP (2014). L5–S1 Achromobacter xylosoxidans infection secondary to oxygen-ozone therapy for the treatment of lumbosacral disc herniation: a case report and review of the literature. Spine.

[12] Pamuk G, Aygun D, Barut K, Kasapcopur O (2015). Achromobacter causing a thrombophlebitis and osteomyelitis combination: a rare cause. BMJ Case Reports.

[13] Shinha T, Oguagha IC (2015). Osteomyelitis caused by *Achromobacter*
*xylosoxidans*. IDCases.

[14] Imani S, Wijetunga A, Shumborski S, O'Leary E (2021). Chronic osteomyelitis caused by *Achromobacter xylosoxidans* following orthopaedic trauma: a case report and review of the literature. IDCases.

[15] Treviño González JL, Reyes Suárez LL, Hernández de León JE (2021). Malignant otitis externa: an updated review. Am J Otolaryngol.

[16] Cohen D, Friedman P (1987). The diagnostic criteria of malignant external otitis. J Laryngol Otol.

[17] Moss WJ, Finegersh A, Narayanan A, Chan JYK (2020). Meta-analysis does not support routine traditional nuclear medicine studies for malignant otitis. Laryngoscope.

[18] Cooper T, Hildrew D, McAfee JS, McCall AA, Branstetter BF, Hirsch BE (2018). Imaging in the diagnosis and management of necrotizing otitis externa: a survey of practice patterns. Otol Neurotol.

[19] Stern Shavit S, Bernstine H, Sopov V, Nageris B, Hilly O (2019). FDG-PET/CT for diagnosis and follow-up of necrotizing (malignant) external otitis. Laryngoscope.

[20] Rodrigues LP, Teixeira VR, Alencar-Silva T, Simonassi-Paiva B, Pereira RW, Pogue R, Carvalho JL (2021). Hallmarks of aging and immunosenescence: connecting the dots. Cytokine Growth Factor Rev.

